# Water-enhanced Removal of Ciprofloxacin from Water by Porous Graphene Hydrogel

**DOI:** 10.1038/srep13578

**Published:** 2015-09-04

**Authors:** Jie Ma, Mingxuan Yang, Fei Yu, Jie Zheng

**Affiliations:** 1 State Key Laboratory of Pollution Control and Resource Reuse, School of Environmental Science and Engineering, Tongji University, 1239 Siping Road, Shanghai 200092, P. R. of China; 2 College of Chemistry and Environmental Engineering, Shanghai Institute of Technology, Shanghai 2001418, China; 3 Department of Chemical and Biomolecular Engineering, The University of Akron, Akron, Ohio, USA 44325

## Abstract

An environmentally benign and efficient hydrothermal reduction method was applied for the preparation of three-dimensional (3D) porous graphene hydrogel (GH) adsorbents. The physicochemical properties of GH granules were systematically characterized by transmission electron microscopy (TEM), X-ray diffraction (XRD), Raman spectra and Brunauer-Emmett-Teller (BET) method. GH granules showed an excellent adsorption capacity (235.6 mg/g) for ciprofloxacin via combined adsorption interaction mechanisms (e.g. π-π EDA interaction, hydrogen bonding, and hydrophobic interaction). Moreover, reducing the size of the hydrogels can significantly accelerate the adsorption process and enhance the removal efficiency of pollutants from aqueous solution. Water (more than 99 wt%) within hydrogels played a key role in enhancing adsorption performance. The GO hydrogels exhibited an excellent adaptability to environmental factors. These findings demonstrate that GH granules are promising adsorbents for the removal of antibiotic pollutants from aqueous solutions.

Antibiotics have been considered as emerging pollutants due to their continuous input and persistence in the aquatic ecosystem even at very low concentrations^1^. Meanwhile, the abuse of antibiotics also results in huge quantities of antibiotic wastewater, by which bacterial resistance was introduced to natural ecosystems^2^. Recent studies have showed that 68 different antibiotics were detected in surface water in China^3^, making the treatment of antibiotic wastewater become a global concern in public health and enviroment. Therefore, a wide range of chemical and physical methodologies have been developed to treat antibiotic wastewater by removing various organic compounds, such as chemical oxidation^4^, biodegradation^5^, adsorption^6^, liquid extraction^7^ and membrane techniques^8^. Among these methods, the adsorption process is regarded as a promising method for the removal of micropollutants because of its simple design, low-cost, high efficiency, and less production of toxic intermediates. So, it is of great importance to develop new types of efficient adsorbents. For a variety of well-known drugs and antibiotics, ciprofloxacin attracts more attention because of its wide use and large production. Hence, we selected ciprofloxacin as a representative target pollutant in this study.

Graphene, a new two-dimensional (2D) carbon nano-material, has been continuously attracting significant research interests because of its unique properties of high mobility of charge carriers, high mechanical strength, super thermal and chemical stability with many potential applications in different fields^9^. Especially, graphene itself owns a theoretical large specific surface area (SSA, 2630 m^2^/g), making it a potential candidate as a high-performance adsorbent^10^. However, aggregation effect, which is caused by its strong van de Waals’ force between 2D graphene sheets during the reduction process, can dramatically decrease the SSA of graphene. And then, its potential applications were restricted as promising adsorbents for organic contaminants. As a result, when these 2D sheets are used as adsorbents, a considerable part of their SSA will be lost. Furthermore, nano-size graphene adsorbent is also very difficult for separation, further increasing application costs in the adsorbent disposition process. Thus it is an important but challenging issue of how to make full use of SSA of graphene , which needs to be solved prior to developing graphene-based adsorbents.

To overcome the SSA restrictions of the graphene, researchers have proposed an efficient strategy by using graphene as a building block, and then integrating it into a desired macroscopic three-dimensional (3D) materials by the self-assembly method, which can efficiently prevent the agglomeration and restacking of graphene sheets, broadening the range of nano-materials in practical applications. Untill now, various methods^11^, including flow-directed assembly^12^, evaporation induced self-assembly^13^, the Langmuir-Blodgett technique^14^, layer-by-layer deposition^15^, hydrothermal reduction^16^, and chemical bonding via crosslinking agents^17^ have been developed to fabricate 3D graphene structures based on the self-assembly of 2D functionalized graphene. Among these techniques, hydrothermal reduction of aqueous graphene oxide (GO) dispersion is a relatively simple, low-cost, and mild method for the synthesis of 3D GO hydrogels. Moreover, the GO hydrogel can be easily separated from the water by filtration or decantation after adsorption, which makes it be a promising adsorbent in water purification^18^,^19^. However, there have been no relevant studies for antibiotics removal using GO hydrogel. Thus, the use of GO hydrogel in adsorption process and its underlying adsorption mechanisms needed be studied from both fundamental and practical viewpoints. Chen *et al.*^20^ recently reported that the hydrogel exhibited remarkably slower adsorption kinetics than other adsorbents, which may be attributed to the slow diffusion of adsorbate molecules in the hydrogel. Slower adsorption rate will definitely limit the practical use of hydrogel, therefore, reducing the size of the hydrogel and agitating the solution may accelerate the diffusion of the adsorbate molecules between the solution and the hydrogel^20^,^21^.

In this study, we applied an environmentally benign and efficient route for the preparation of granule GO hydrogels by a one-step hydrothermal reduction of aqueous GO dispersion. GH was first used as an adsorbent material in granule scale for removing ciprofloxacin from aqueous solutions and its excellent adsorption properties and faster adsorption rate have been achieved. Moreover, the adsorption removal mechanism of ciprofloxacin and its influence factors were investigated through comparative trials, indicating that GH possesses a wide adaption range in practical applications. Therefore, GH could serve as a promising adsorbent for the removal of antibiotic pollutants from aqueous solutions.

## Materials and Methods

### Materials

All chemicals were purchased from Sinopharm Chemical Reagent Co., Ltd (Shanghai, China) in analytical purity and used in the experiments directly without any further purification. All solutions were prepared using deionized water.

### Preparation of adsorbents

The graphite oxide was prepared according to a modified Hummer’s method^22^. Graphite oxide was dispersed in deionized water and sonicated in an ultrasound bath for 6 h to obtain GO aqueous dispersion (2.0 mg/mL). Then, ascorbic acid was added to get a concentration of 2.0 mg/mL. After ultrasonic dispersion for 15 min to dissolve the ascorbic acid, 0.5 mL as-prepared dispersion was loaded in a glass vial and heated at 80 °C for 12 h without any disturbance^23^. After the chemical reduction, the obtained GH granules were placed in deionized water for 12 h to remove the excess ascorbic acid. Then granule GO aerogels (GA) have been achieved by a freeze-drying process using GH.

### Batch adsorption experiments

Batch adsorption experiments were carried out in 25 mL volumetric capacity batch bottles with 5 GH granules and 20 mL ciprofloxacin solution of different initial concentrations. Timing of the sorption period started as soon as the solution was poured into the bottle. Sample bottles were shaken on a shaker (TS-2102C, Shanghai Tensuclab Instruments Manufacturing Co., Ltd., China) and operated at a constant temperature of 25 °C and 150 rpm for 120 h. The blank experiments without the addition of adsorbents were conducted to ensure that the decrease in the concentration was actually due to the adsorption, rather than other factors. After adsorption ciprofloxacin samples were filtered and diluted for UV-Vis measurements.

Kinetic studies were performed at a constant temperature of 25 °C and 150 rpm with 50 mg/L initial concentration of ciprofloxacin solutions with different adsorption time. The ionic strength experiments were conducted in 100 mg/L ciprofloxacin solutions with varying concentrations of NaCl solution (0, 0.001, 0.01, 0.1, 0.5, 1.0 mol/L). The effect of solution pH on ciprofloxacin removal was studied in the range of 2–12 with 100 mg/L initial concentrations of ciprofloxacin solutions. The initial pH values of all the solutions were adjusted using 0.1 mol/L HCl or 0.1 mol/L NaOH solution with desired concentrations.

The absorption capacity of ciprofloxacin (q_e_, mg/g) was calculated as follows


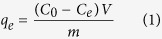


where C_0_ and C_e_ are the initial and residual concentrations of ciprofloxacin (mg/L), V is the initial solution volume (L), and m is the adsorbent weight (g).

### Characterization methods

The microstructure and morphology of the samples from each step were observed by transmission electron microscopy (TEM, JEOL2010F, 200 kV) equipped with an energy dispersive spectrometer (EDS) and field-emission scanning electron microscopy (SEM, Hitachi S-4800). Raman spectroscopy (JOBIN-YVON T64000) was used to characterize further the structural integrity of the graphene oxide and aerogels. X-ray diffraction (XRD) experiments were conducted on the specimens to study the structural phases and average size of the aerogels using a Siemens D5000 X-ray diffractometer (Cu Kα radiation, λ = 1.5406 Å) over a range of 10–90 operated at 40 mA and 40 kV, with a scan rate of 1°/min and a step size of 0.02°. The concentration of ciprofloxacin was determined using UV-V is spectrophotometer (Techcomp UV2310 II) at wavelengths of 270 nm. The SSA and pore size distribution of adsorbents were calculated from the adsorption/desorption isotherms of N_2_ at 77 K by a BELSORP instrument (BEL, Japan, Inc.).

## Results

### Characterization of GH

**Figure 1:**
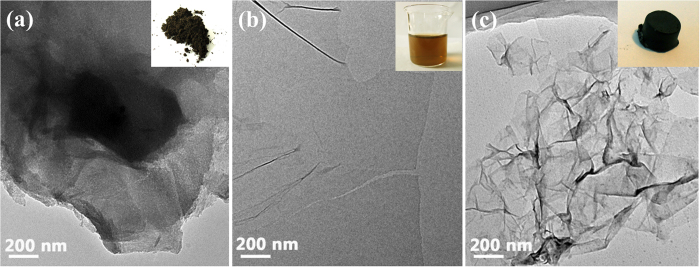
TEM images of Graphite oxide (a), Graphene oxide (b), and GO hydrogel (c).

**Figure 2:**
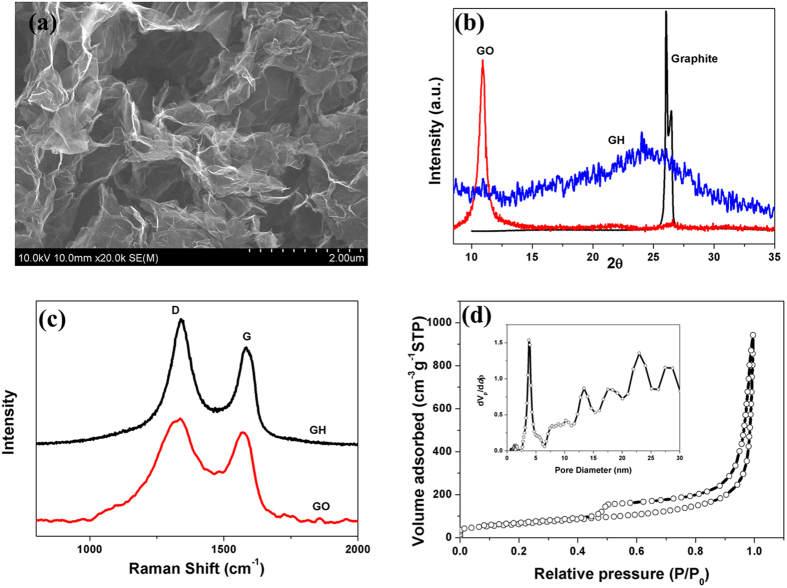
SEM images (a) of GH, XRD patterns (b) of graphite, GO and GH, Raman spectra (c) of GO and GH, and N_2_ adsorption/desorption isotherms and pore size distribution (d) of GA granules.

Figure 1 displays the TEM images of GO hydrogel formation process. Graphite oxide sheets showed tight stack vertically (Fig. 1a). After ultrasonic separation, graphene oxide sheets were separated from each other due to the abundant function groups and electrostatic repulsion between GO sheets. The thickness of GO sheet was ~0.55 nm (Fig. 1b). Then reduction process helped to remove oxygen-contained functional groups, resulting in the formation of hydrophobic graphene and restoration of the conjugated structure^16^. The restored conjugated structure of dispersed graphene sheets provided a greatly increased amount of π-π stacking sites to overlap partially at the edge of each other^24^,^25^ (Fig. 1c). The porous structure consisting of graphene nanosheets stack can be observed from SEM images of GH , as shown in Fig. 2a.

XRD patterns of graphite, GO and GH samples were shown in Fig. 2b. For GO sample, the sharp peak at about 2θ = 10.9° was attributed to the reflection of stacked GO sheets, corresponding to an interlayer spacing of ~0.81 nm for the presence of oxygen-contained functional groups and intercalated water molecules. After the chemical reduction and self-assembly process, a new broad peak appeared at about 2θ = 24.2°, corresponding to an interlayer spacing of ~0.37 nm, larger than that of natural graphite (2θ = 26.2°, d-spacing of 0.33 nm). This indicates the poor ordering of graphene sheets along their stacking direction and the formation of the π-π stacking between graphene sheets, because of the elimination of oxygen-contained functional groups on the GO by the hydrothermal process^11^,^24^.

The Raman results were shown in Fig. 2c. In the Raman spectra, the D peak at 1244 cm^−1^ was induced by the defective structures of carbon material, while the G peak at 1603 cm^−1^ was related to E_2g_ graphite mode, reflecting good graphitization of carbon material. Hence, the intensity ratio of the D to the G peaks (I_D_/I_G_) reflects the defective extent and the structure quality of graphene semi-quantitatively. The greater the intensity ratio of the sample, the poorer the graphite structure of the carbon material owns. After the chemical reduction and self-assembly process, the I_D_/I_G_ intensity ratio of GO hydrogel (1.26) was higher than that of the GO (1.16). This change suggests a decrease in the average size of the sp^2^ domains upon reduction of GO, and can be explained by that new graphitic domains were created with smaller sizes but with more quantities as compared to the ones present in GO^26^. Besides, the presence of unrepaired defects that remained after the removal of large amounts of oxygen-contained functional groups can also result in the increase of I_D_/I_G_ intensity ratios of Raman spectra^27^.

The N_2_ adsorption/desorption isotherms of GA is presented in Fig. 2d and the detailed features of meso-pore analyzed by the DFT method are presented in Fig. 2d and Table 1. The SSA of GA is ~231.38 m^2^/g, much less than theoretical SSA, indicating the agglomeration and restacking of graphene sheets during freeze drying. Besides, GH granules exhibited mainly mesoporous structures with pore diameter range of 0 ~ 30 nm.

### Adsorption kinetics

**Figure 3:**
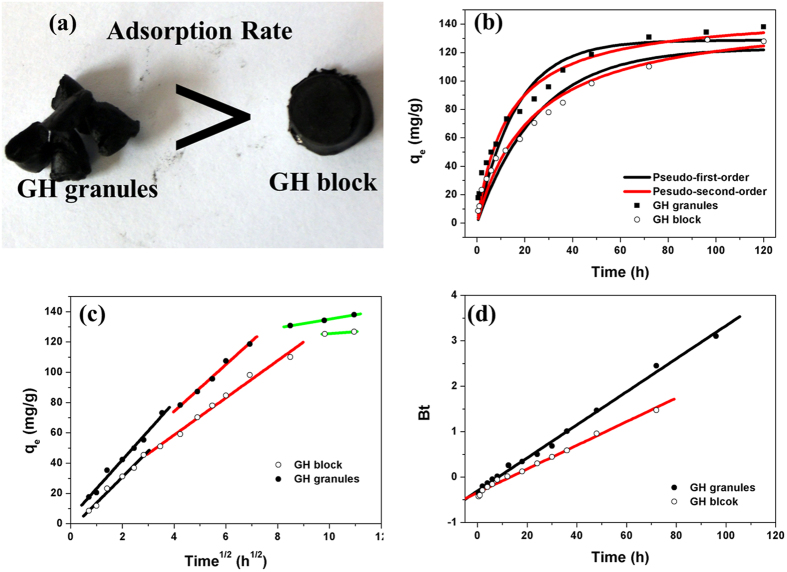
Comparison of macrostructure (a), kinetic curves, pseudo-first-order, pseudo-second-order model (b), Weber-Morris model (c) and Boyd model (d) of GH granules and GH block (ciprofloxacin concentration = 50 mg/L).

Adsorption is a physicochemical process that involves the mass transfer of solutes from the liquid phase to the adsorbent surface. To study the adsorption kinetics of GH block and GH granules, ciprofloxacin solution with initial concentrations of 50 mg/L was used for adsorption process under the fixed GO amount conditions. As shown in Fig. 3b, the adsorption process of ciprofloxacin by GH granules was much faster than traditional GH block, exhibited a rapid removal rate at the initial period (~36 h), and reached 75% of maximum adsorption capacity. Moreover, removal rate became slow and stagnate as contact time (from ~36 to ~120 h), and nearly reached equilibrium after approximately 120 h of the experiment. The adsorption rate of GH is significantly limited by the diffusion of adsorbate molecules through the hydrogel. However, aerogels showed a much faster adsorption rate and lower adsorption capacity than hydrogels(Fig. S1, S2, S3).

To understand the characteristics of the adsorption process, the transient behavior of the adsorption process was analyzed using different kinetic models. Pseudo-first-order (PF) and pseudo-second-order (PS) kinetic models that originate from chemical reaction kinetics were applied to fit experimental data obtained from batch experiments (Fig. 3b). The kinetic parameters and the determination coefficients (R^2^) were determined by nonlinear regression and given in Table 2. It can be seen that the PS kinetic curves gave a better fit to the experimental kinetic data for both GH and GA than the PF model, indicating the PS kinetic model is more appropriate to describe the adsorption behavior of ciprofloxacin onto GH and GA and the chemical adsorption occurred in the process. Meanwhile, K parameter of GH granules was larger than GH block, indicating reducing the size of the hydrogel is an efficient way to accelerate the adsorption process^28^.

The adsorption rate is controlled by outer diffusion or inner diffusion or both. In that case, the Weber-Morris model was applied to help determine the actual rate-controlling step in ciprofloxacin sorption process. Plots of q_t_ against t_1/2_ and the linear regression of q_t_ versus t_1/2_ are shown in Fig. 3c, and the corresponding kinetic parameters are listed in Table S1. It was also observed that the adsorption process of GH block and GH granules can be divided into three stages according to Weber-Morris model where the slope corresponds to the adsorption rate. At the first stage, the regression line with little intercepts almost passed through the origin, suggesting that the intra-particle diffusion is not the sole rate-controlling step, instead played a predominant role at the initial stage. The sharper slope was attributed to the diffusion of ciprofloxacin through the solution to the external surface of hydrogel, or the boundary layer diffusion of ciprofloxacin. GH granules possess faster adsorption rate mainly due to its longer first stage than GH block. The second stage implied the gradual adsorption stage, where intra-particle diffusion was rate-controlling. As the contact time increases, the effect, that external mass transfer played in adsorption rate-controlling, becomes more and more evident for the intercepts of fit linear increased. The intra-particle diffusion slowed down at the third stage due to the extremely low ciprofloxacin concentration left in the solution. The overall adsorption process may be jointly controlled by external mass transfer and intra-particle diffusion, and intra-particle diffusion played a predominant role at the first stage^29^.

The adsorption kinetic data were further analyzed by the Boyd model to determine the actual rate-controlling step involved in the ciprofloxacin sorption process. The calculated Bt values were plotted against time t as shown in Fig. 3d. The regression lines in Fig. 3d do not pass through the origin, confirming the involvement of intra-particle diffusion in the entire GH adsorption process^30^. These results again confirm the rate-controlling mechanism of adsorption stated in Weber-Morris kinetic model studies. Therefore, the adsorption rate is controlled by both external mass transfer and intra-particle diffusion^28^. Besides, the slope of GH granules was sharper than traditional GH block, indicating a faster adsorption process.

### Adsorption Isotherms

**Figure 4:**
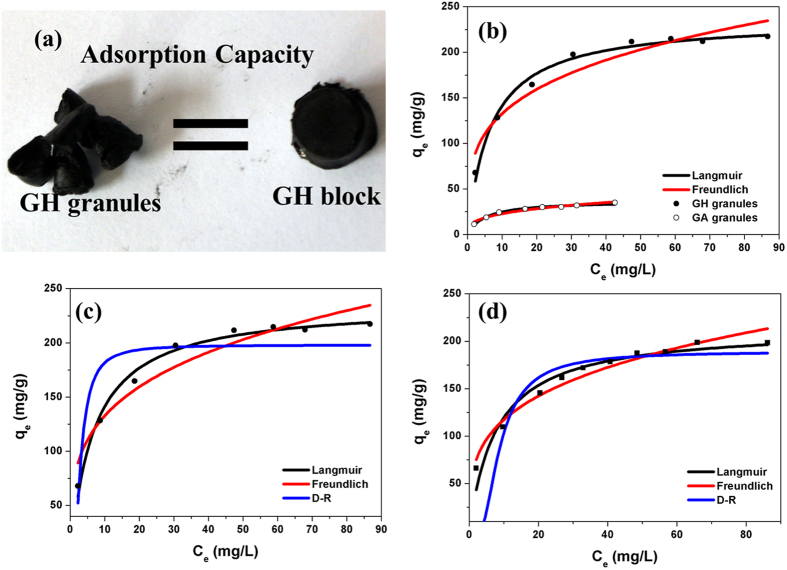
Macrostructure comparison (a) of GH granules and GH block, Equilibrium adsorption isotherms of GH granules and GA granules (b), GH granules (c) and GH block (d).

To learn more about adsorption characteristics of ciprofloxacin onto GH and GA granules, traditional adsorption equilibrium isotherms experiments were designed. Figure 4 shows the equilibrium isotherms data for adsorption of ciprofloxacin onto GH granules, GH block and GA granules. The classic isotherm models of Langmuir and Freundlich were used to fit the experimental equilibrium adsorption data and analyze equilibrium adsorption characteristics. The isotherms parameters obtained from the nonlinear fit by both models are listed in Table 3 along with determination coefficients (R^2^). Based on the determination coefficient shown in Table 3, both models showed a good fit with adsorption data (R^2^ > 0.90) and GH granules showed almost the same adsorption capacity as GH block, indicating that reducing the size of the hydrogel has almost no influence on adsorption capacity. The R_L_ value of ciprofloxacin adsorption is ~0.063 for GH granules and ~0.199 for GA granules, indicating that the adsorption of ciprofloxacin onto GH and GA are a favorable process. For GH granules, Langmuir isotherm model showed a relatively better fit than Freundlich isotherm model, indicating specific homogenous sites within the adsorbent are involved^28^. But GA granules showed a slight difference between Langmuir isotherm model fit and Freundlich isotherm model fit (Fig. S4), suggesting highly heterogeneous distribution of sorption energy and existence of the intermolecular interactions that occur between ciprofloxacin and GA granules. This result can be understood from the following three aspects: (a) the presence of bound water in hydrogels changed the surface characteristics of aerogels; (b) more oxygen-containing groups are involved and provide more specific homogenous sites. (c) during the preparation of the aerogels by dry-freezing, the volume of water expands to some extents, which may destroy 3D porous hydrogel structure and decrease SSA, making adsorption site more heterogeneous.

To deeply understand the adsorption mechanism, Dubinin-Radushkevich (D-R) isotherm model was chosen to distinguish the physical and chemical adsorption of ciprofloxacin onto graphene hydrogels and aerogels. The isotherm constants, E and determination coefficients are calculated and presented in Table 3. The values of E (mean energy of adsorption) exceed ~16 kJ/mol for both GH and GA, which suggests that chemical adsorption is dominating in the adsorption process between ciprofloxacin and adsorbents^28^.

Table 4 presents a comparison of the maximum adsorption capacities (q_m_) of ciprofloxacin onto various adsorbents. It is clear from this comparison table that the GH gave a higher adsorption capacity for ciprofloxacin than many other carbon-based adsorbents as reported. The results suggest that the GH granules adsorbents hold great potential for ciprofloxacin removal from aqueous solutions.

### Effect of pH and ionic strength

**Figure 5:**
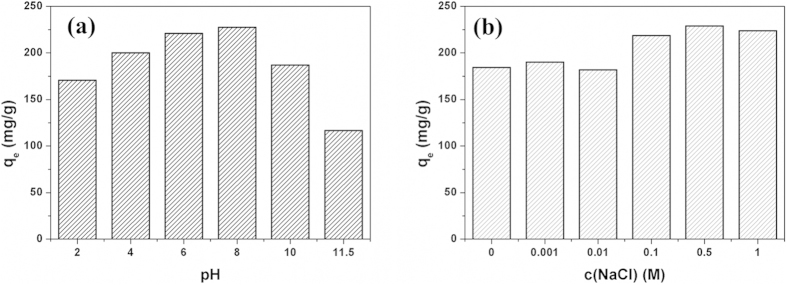
Effect of pH (a) and ionic strength (b) on the adsorption capacity of ciprofloxacin on GH granules (ciprofloxacin concentration = 100 mg/L).

Figure 5a shows the effect of pH on the adsorption capacity of ciprofloxacin on GH granules. The ciprofloxacin removal rate increased as pH and the highest ciprofloxacin removal rate was observed at pH 8.0, above which it decreased. The effect of solution pH on the observed removal behavior of ciprofloxacin can be attributed to a combination of pH-dependent speciation of ciprofloxacin and surface charge characteristics of the hydrogel. Since ciprofloxacin is a zwitterionic compound showing two pKa values (pK_a1_ = 6.1; pK_a2_ = 8.7), ciprofloxacin is presented as a cationic form at pH < 6.1, whereas it has an anionic form at pH > 8.7. When the pH value was very low, the ciprofloxacin existed mainly in a cationic form. Meanwhile, the surface of the hydrogel became positively charged, thus repelling the overly promoted ciprofloxacin and resulting in the lower adsorption. As pH increased, the decrease in the cationic form of ciprofloxacin weakens electrostatic repulsion effect, leading to the rise of adsorption capacity. At 6.1 < pH < 8.7, ciprofloxacin is zwitterionic and in favor of adsorption. Similarly, when the solution pH was above 8.0, the surface of the hydrogel became negatively charged, strongly repelling the ciprofloxacin in anionic form and resulting in lower adsorption^31^.

Since sodium chloride is a common salt existed in surface water, the effect of ionic strength on the adsorption of ciprofloxacin onto hydrogel was further tested by NaCl of different concentrations, and the results were shown in Fig. 5b. The adsorption capacity of GH remained a plateau at a low salt concentration and increased when ionic strength was high enough(>0.1 M). Ionic strength affects the adsorption process by influencing the activity coefficients of OH, H_3_O^+^, and specifically the adsorbate ions. Theoretically, when the electrostatic forces between the adsorbent surface and adsorbate ions are attractive, an increase in ionic strength will increase the adsorption capacity. Conversely, when the electrostatic attraction is repulsive, an increase in ionic strength will increase adsorption^28^. Since ciprofloxacin remains zwitterionic at neutral pH and the electrostatic interactions between the ciprofloxacin and hydrogels were almost negligible. Thus there is no significant adsorption difference under low NaCl conditions(0, 0.001, 0.01 and 0.1 M). However, when ionic strength is large enough, the adsorption capacity of ciprofloxacin onto hydrogel increased with the increase of ionic strength in the solution. This result may be explained by that the NaCl addition increase the osmotic pressure of adsorbate, which promotes more ciprofloxacin to transfer into micropores filled with water, thus increasing the adsorption capacity.

## Discussion

**Figure 6:**
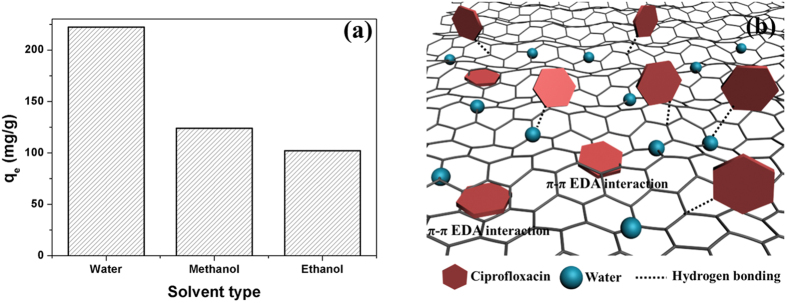
Adsorption capacity comparison (a) of GH granules in different solvents, and adsorption removal mechanism (b) of ciprofloxacin by GH granules.

In order to investigate the ciprofloxacin adsorption mechanism of GH granules, comparative trials were designed by using methanol and ethanol as solvents, respectively and the GH granules synthesized above were soaked in either methanol or ethanol to replace water. Comparative trial results were shown in Fig. 6a. Hydrogels in water showed much higher adsorption capacity than other conditions, while hydrogels in methanol adsorbed more contaminants than in ethanol. The large adsorption difference can be attributed to the significant role of water in the adsorption process. Firstly, alcohol solvents can not provide strong hydrophobic interactions between the carbon surface and ciprofloxacin. Besides, water contains more hydrogen bonds to associate with ciprofloxacin than other solvents.

As shown in Fig. 6b, GH granules offer great advantages over GA granules for ciprofloxacin removal. The only difference between GH granules and GA granules is the existence of water. So it is easy to infer that water has large effects on the adsorption process. Thus, the adsorption removal mechanism of ciprofloxacin by hydrogels mainly involves three removal pathways occurring simultaneously: (a) π-π electron donor-acceptor (EDA) interaction, (b) hydrogen bonding, and (c) hydrophobic interaction^32^,^33^,^34^ between hydrogel and ciprofloxacin. A number of studies have shown that π-π electron donor-acceptor (EDA) interaction has been treated as one of the predominant driving forces for the sorption of organic chemicals with benzene rings on carbon surface^10^,^34^,^35^. The benzene rings with fluorine group and N-heteroaromatic ring on ciprofloxacin molecules can function as a π-electron-acceptor due to the strong electron withdrawing ability of N and F. Meanwhile, various oxygen-containing groups, -OH and -COOH, exist on the surface of graphene oxide, making graphene oxide more hydrophilic. The -OH groups on graphene surface make the benzene rings as π-electron donors, whereas the -COOH groups make the benzene rings as π-electron acceptors to depress π-π bonds^36^. In the chemical reduction process, most of -COOH groups were removed due to its high oxidability. Thus residual -OH groups on graphene surface make the benzene ring the π-electron-rich ring, which plays an important role in π-π electron donor-acceptor interaction.

Hydrogen bond existence has been widely proved in the process of polar organic pollutants sorption^33^,^37^. Previous studies have found that ciprofloxacin molecule with two -C = O and one -OH groups can form hydrogen bond with the surface-oxygen of carbon material, as well as with the functional groups on the surface. Functional groups of organic chemicals such as -NH and -OH act as hydrogen bonding donors to form hydrogen bonds with the graphene sheets, where the benzene rings serve as hydrogen-bonding acceptors. Similarly, polar functional groups in ciprofloxacin such as F, N, O can also act as hydrogen-bonding acceptors and form hydrogen bonding with -OH on graphene. The additive effect of multiple hydrogen-bonding between ciprofloxacin and adsorbents further improves adsorption affinity.

However, for traditional carbon materials such as carbon nanotubes and graphene, the effects of hydrogen-bonding interaction on adsorption of solutes may not be significant due to the lack of functional groups on their surface. Moreover, hydrophilic functional groups of carbon materials will form hydrogen bonds with water molecules, leading to the competitive sorption of water with organic solutes. However, for hydrogels, another major character is its high water content, which occupy more than 99 wt.%, like active sludge. The massive bound water in the hydrogel can be treated as surface functional groups, providing a large amount of -OH for the hydrogel. In this case, bound water in hydrogel acted as another type of adsorbent, which offers more hydrogen bonding donors and acceptors and thus greatly enhance its adsorption capacity for ciprofloxacin. Another possible mechanism is a hydrophobic interaction between the carbon surface and ciprofloxacin molecule. In aqueous solution, the adsorbate of higher hydrophobicity has stronger tendency to be adsorbed on the carbon surface or in the pores^38^. The removal of oxygen-containing groups on GO in reduction process increased its hydrophobicity, which provides many hydrophobic sites to interact with hydrophobic molecules. Hydrogels in alcohols solvents cannot adsorb contaminants via hydrophobic interaction. Hydrogels in water show much higher adsorption capacity than other conditions, which could prove the existence of hydrophobic interaction. As such, the contribution of hydrophobic interaction to ciprofloxacin sorption can not be excluded, despite the lack of substantial evidence for this process^32^.

## Conclusions

In this work, we have demonstrated a facile solution processable method for the synthesis of 3D porous graphene hydrogels using ascorbic acid as a reducing agent. The resulting GH granules were used as efficient adsorbents for ciprofloxacin removal in aqueous solutions. Collective experimental data showed that GH granules exhibited excellent ciprofloxacin adsorption capacity of 235.6 mg, which can be attributed to multiple adsorption interaction mechanisms (e.g. π-π EDA interaction, hydrogen bonding, hydrophobic interaction) between hydrogel and ciprofloxacin. Reduction of hydrogel sizes enables to accelerate the adsorption process and thus enhance the removal efficiency of pollutants. Moreover, we found that water can provide more hydrogen bonds to associate with ciprofloxacin than other solvents, water in hydrogels promoted the adsorption process, and GO hydrogels own excellent adaptability to environmental factors. These findings indicate that GO hydrogels serve as a promising adsorbent for removal of antibiotic pollutants from aqueous solutions.

## Additional Information

**How to cite this article**: Ma, J. *et al.* Water-enhanced Removal of Ciprofloxacin from Water by Porous Graphene Hydrogel. *Sci. Rep.* **5**, 13578; doi: 10.1038/srep13578 (2015).

## Supplementary Material

Supplementary Information

## Figures and Tables

**Table 1:** Detailed physical features of GA aerogels.

Samples	SSA	PV	micro-PV	ESA	ISA
GA	231.38	1.457	0.016	202.55	28.83

SSA = specific surface area (m^2^/g); ISA = internal surface area (m^2^/g); ESA = external surface area (m^2^/g); PV = pore volume (cm^3^/g).

**Table 2:** Kinetic parameters of pseudo first-order and second-order adsorption kinetic models and Boyd model for ciprofloxacin on GH granules, GH block and GA granules.

Adsorbents	Pseudo-first-order Model			Pseudo-second-order Model			Boyd Model		
	K_1_(h^−1^)	q_1_(mg/g)	R^2^	K_2_(h^−1^)	q_2_(mg/g)	R^2^	K_B_	b	R^2^
GH granules	0.0265	128.706	0.924	0.0169	123.090	0.963	0.036	−0.306	0.995
GH block	0.0169	123.090	0.946	0.0003	148.867	0.970	0.026	−0.344	0.991
GA granules	0.2294	23.547	0.957	0.0221	27.265	0.935	0.969	−1.010	0.965

**Table 3:** Langmuir, Freundlich, and Dubinin-Radushkevich Isotherm Parameters of GH granules, GH block and GA granules.

Adsorbents	Langmuir model				Freundlich model			Dubinin-Radushkevich model			
	K_L_ (1/mg)	q_m_ (mg/g)	R_L_	R^2^	K_F_	n	R^2^	B(mol/KJ^2^,×10^−6^)	q_m_ (mg/g)	E (KJ/mol)	R^2^
GH granules	0.150	235.64	0.063	0.985	72.390	3.794	0.919	1.544	198.06	569.1	0.742
GH block	0.126	214.46	0.073	0.949	61.99	3.600	0.963	1.757	189.15	533.4	0.745
GA granules	0.201	37.14	0.199	0.981	11.235	3.241	0.960	1.182	30.03	650.4	0.762

**Table 4:** Comparison of the adsorption capacities of ciprofloxacin onto various adsorbents.

Adsorbents	Adsorption capacity (mg/g)	Refs
GH	235.64	This study
Activated carbon	231 ± 6	25
Carbon xerogel	112 ± 8	25
Carbon nanotubes	135 ± 2	25
Chemically prepared carbon	104.2–133.3	26
Nano-Fe_3_O_4_	12–37	27
Graphene oxide/calcium alginate fibers	66.25	28
Graphene oxide/magnetite composites (RGO-M)	10.91	29
Fe_3_O_4_/C	74.68	30
Modified Carbon nanotubes	106–138	31
