# Supplementary material for: Water-enhanced Removal of Ciprofloxacin from Water by Porous Graphene Hydrogel
Source: Sci Rep. 2015 Sep 4;5:13578. doi: 10.1038/srep13578 (PMC4559748; doi:10.1038/srep13578)
Supplement: Supplementary Information [file srep13578-s1.pdf]

Support Information

Water-enhanced Removal of Ciprofloxacin from Water by Porous Graphene Hydrogel

Jie Ma[a]*, Mingxuan Yang[a], Fei Yu[a,b]*, Jie Zheng[c]

[a*] State Key Laboratory of Pollution Control and Resource Reuse, School of Environmental Science and Engineering, Tongji University, 1239 Siping Road, Shanghai 200092, P. R. of China.

Tel: 86-21-6598 1831; E-mail: jma@tongji.edu.cn

[b*] College of Chemistry and Environmental Engineering, Shanghai Institute of Technology, Shanghai 2001418, China, Tel: 86-21-6087 3182; E-mail: fyu@vip.163.com

[c] Department of Chemical and Biomolecular Engineering, The University of Akron, Akron, Ohio, USA 44325.

Analysis Method.

Isotherm Model.

Langmuir Model.

The form of the Langmuir isotherm can be represented by the following equation:

$$q_e = q_m \frac{K_L C_e}{1 + K_L C_e} \qquad (1)$$

where q_e is the absorption capacity of ciprofloxacin (mg/g), C_e denotes the equilibrium concentration of ciprofloxacin in solution (mg/L); K_L represents the Langmuir constant (L/mg) that relates to the affinity of binding sites, and q_m is a theoretical limit of adsorption capacity when the monolayer surface is fully covered with ciprofloxacin molecules to assist in the comparison of adsorption performance (mg/g). Furthermore, the effect of the isotherm shape was studied to understantwhether an adsorption system is favorable or not. Another important parameter, R_L, called the separation factor or equilibrium parameter, which can be used to determine the feasibility of adsorption in a given concentration range over adsorbent, was also evaluated from the relation

$$R_L = \frac{1}{1 + K_L C_0} \qquad (2)$$

where K_L is the Langmuir adsorption constant (l/mg) and C_0 is the initial ciprofloxacin concentration (20 mg/L). Ho and McKay established that (1) $0 < R_L < 1$ for favorable adsorption; (2) $R_L > 1$ for unfavorable adsorption;

(3) R_L=1 for linear adsorption; and (4) R_L=0 for irreversible adsorption.

Freundlich Model.

The Freundlich isotherm model has the following form

$$q_e = K_F C_e^{1/n} \qquad (3)$$

where q_e is the absorption capacity of ciprofloxacin (mg/g), C_e is the equilibrium concentration of ciprofloxacin in solution (mg/L); K_F and n are the Freundlich constants, which represent the adsorption capacity and the adsorption strength, respectively. The magnitude of 1/n quantifies the favorability of adsorption and the degree of heterogeneity of the adsorbent surface.

Dubinin−Radushkevich (D-R) Model.

The D-R isotherm model has the following form:

$$\ln q_e = \ln q_m - B£^2 \qquad (4)$$

where B is a constant related to the mean free energy of adsorption (mol^2/kJ^2), q_m is the theoretical saturation capacity, and ε is the Polanyi potential, which is equal to

$$£ = RT \ln\left(1 + \frac{1}{C_e}\right) \qquad (5)$$

where R (J/mol·K) is the gas constant and T (K) is the absolute temperature. For D-R isotherm model, from B values the mean energy of adsorption. The mean energy of adsorption (E) is the free energy change when one mole of the ion is transferred from infinity in the solution to the surface of the solid. E can be calculated using the relation

$$E = \frac{1}{\sqrt{-2B}} \quad (6)$$

Kinetic Model.

Pseudo-First-Order and Pseudo-Second-Order Model.

The linear form of pseudo-first-order rate equation is

$$\ln\left(q_e - q_t\right) = \ln q_e - \frac{K_1}{t} \quad (7)$$

where q_e and q_t are the amounts of ciprofloxacin adsorbed (mg/g) at equilibrium and time t (h) respectively; K_1 is the rate constant of the pseudo first-order kinetic model (h^{-1}).

A linear form of pseudo second-order kinetic model is express by eq

$$\frac{t}{q_t} = \frac{1}{k_2 q_e^2} + \frac{t}{q_e} \quad (8)$$

where k_2 is the rate constant(g mg-1min-1) of pseudo second-order kinetic model for adsorption.

Weber−Morris Kinetics Model.

Intra-particle mass transfer diffusion model proposed by Weber and Morris can be written as follows

$$q_t = k_i t^{1/2} + C \quad (9)$$

where C (mg/g) is the intercept and k_i is the intra-particle diffusion rate constant ($g/(mgh^{-0.5})$) for adsorption.

Boyd Model. Boyd model has the following form

$$Bt = -\ln\left(1 - \frac{q_t}{q_e}\right) - 0.4977 \qquad (10)$$

where q_t and q_e are the amounts of ciprofloxacin adsorbed on the adsorbent (mg/g) at time t (h) and at equilibrium time (h) respectively; B = 2Di/r2 (Di is the effective diffusion coefficient of the adsorbate and r is the radius of adsorbent particles assumed to be spherical).

Table S1 Kinetic parameters of Weber-Morris model for ciprofloxacin on GH granules and GH block

Adsorbents	Stage1			Stage2			Stage3		
	K_1(g^{-1} mg^{-1} h^{-1})	C_1 (mg/g)	R^2	K_1(g^{-1} mg^{-1} h^{-1})	C_1 (mg/g)	R^2	K_1(g^{-1} mg^{-1} h^{-1})	C_1 (mg/g)	R^2
GH granules	19.056	4.083	0.981	15.467	12.290	0.988	2.926	105.825	0.992
GH block	17.142	-3.508	0.984	12.279	9.471	0.982	1.283	112.699	-

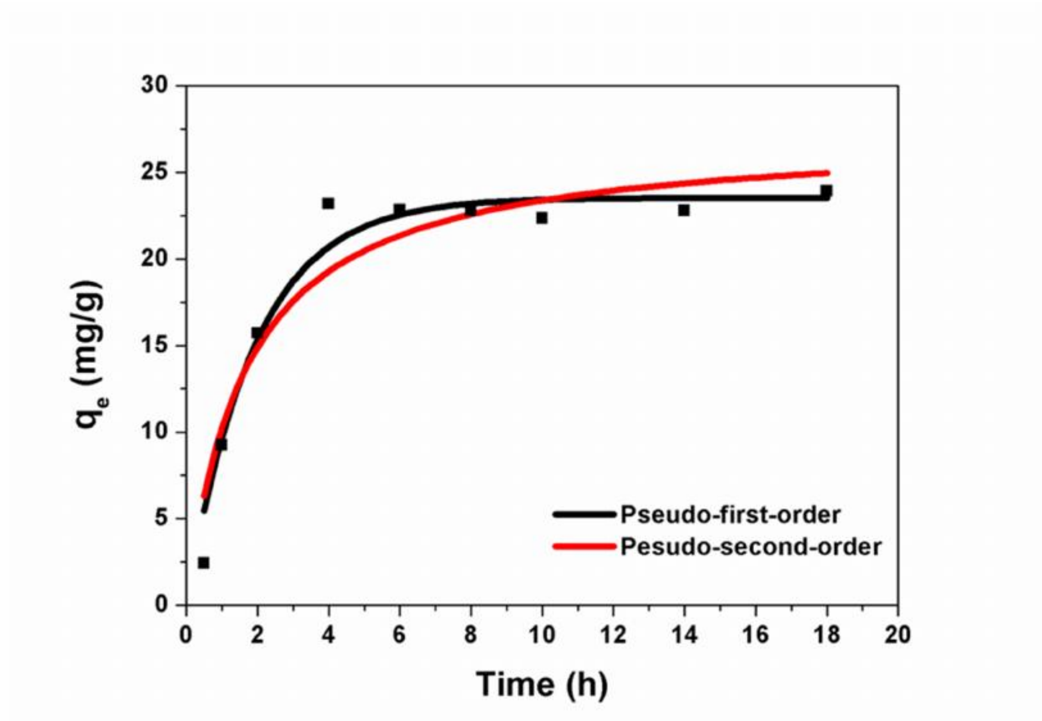

Fig.S1 Kinetic curves, pseudo-first-order, pseudo-second-order model of GA granules

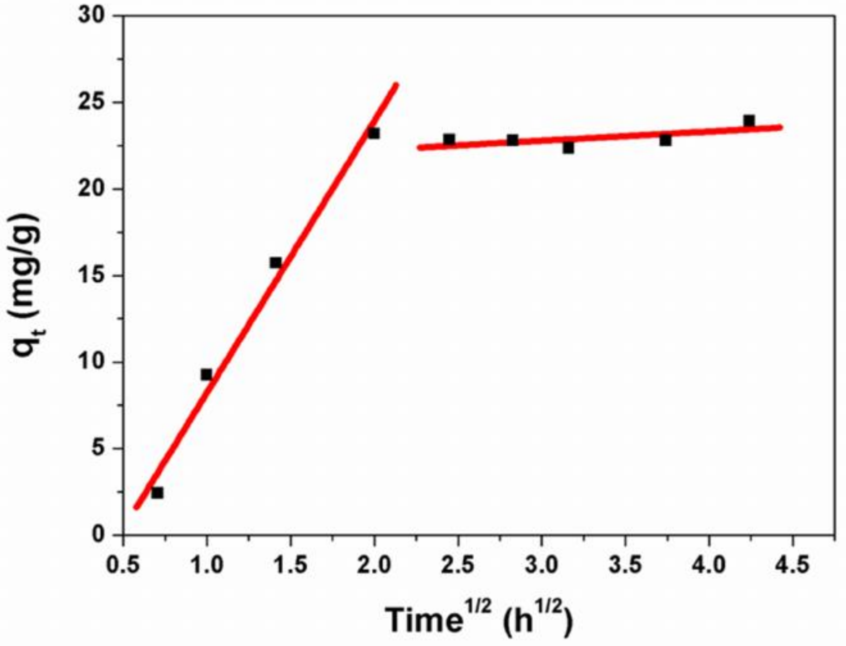

Fig.S2 Weber-Morris model of GA granules

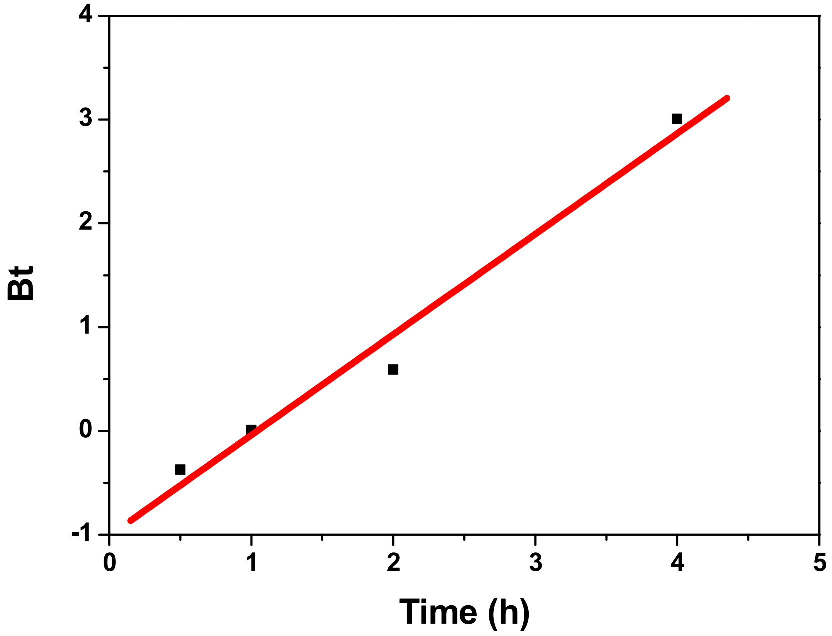

Fig.S3 Boyd model of GA granules

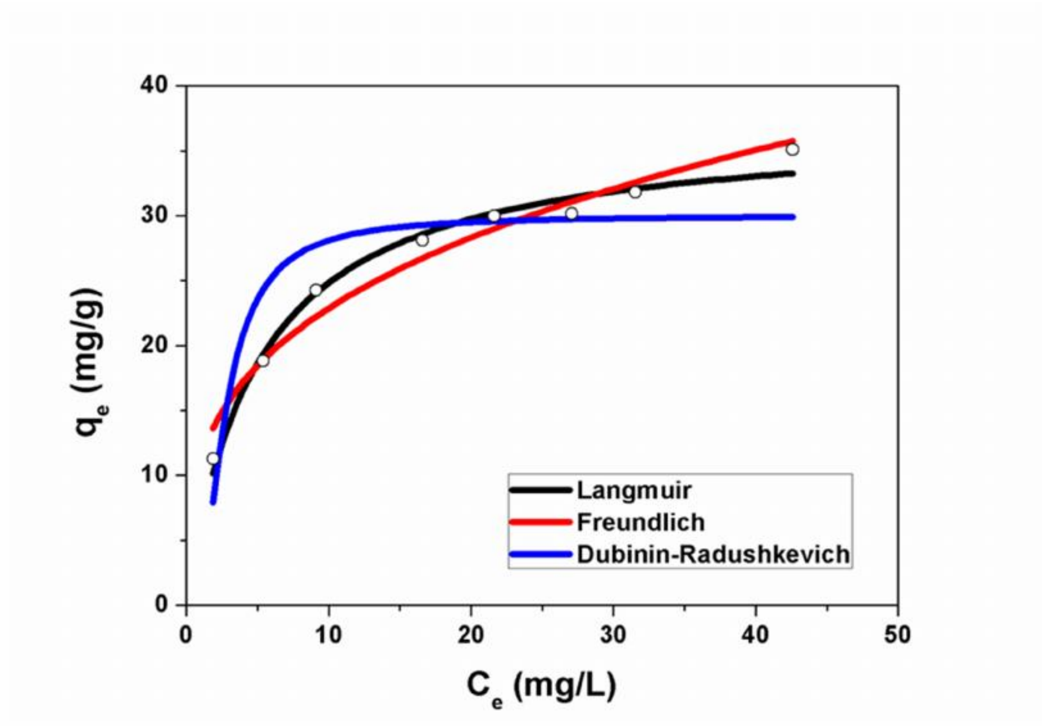

Fig.S4 Equilibrium adsorption isotherms of GA granules
